# Next-Generation-Sequencing-Based Simple Sequence Repeat (SSR) Marker Development and Linkage Mapping in Lentil (*Lens culinaris* L.)

**DOI:** 10.3390/life13071579

**Published:** 2023-07-18

**Authors:** Mustafa Topu, Uğur Sesiz, Harun Bektaş, Faruk Toklu, Hakan Özkan

**Affiliations:** 1 Department of Biotechnology, Institute of Natural and Applied Sciences, Çukurova University, 01330 Adana, Turkey; mustafatopu@gmail.com (M.T.); fapet@cu.edu.tr (F.T.); 2 Department of Field Crops, Faculty of Agriculture, Çukurova University, 01330 Adana, Turkey; usesiz47@gmail.com; 3 Department of Field Crops, Faculty of Agriculture, Şırnak University, 73300 Şırnak, Turkey; 4 Department of Agricultural Biotechnology, Faculty of Agriculture, Siirt University, 56100 Siirt, Turkey; bektasharun@gmail.com

**Keywords:** lentil, *Lens culinaris*, SSR development, linkage map, next-generation sequencing

## Abstract

**Simple Summary:**

Although lentil is not as popular as other legumes, it is a climate-resilient legume crop because of its high protein content, nitrogen fixation, and abiotic stress tolerance ability. Even though it can be grown on almost every continent and is distributed globally, the use of existing genetic diversity in marker-assisted selection is still limited. In this study, novel SSR markers needed in lentil were identified using a next-generation sequencing approach. In this study, we created a ready-to-use SSR library for genetic diversity studies and breeding and evaluated the effectiveness of the obtained SSR markers in a recombinant inbred line (RIL) population.

**Abstract:**

Simple sequence repeats (SSRs) are highly versatile markers in genetic diversity analysis and plant breeding, making them widely applicable. They hold potential in lentil (*Lens culinaris*) breeding for genetic diversity analysis, marker-assisted selection (MAS), and linkage mapping. However, the availability and diversity of SSR markers in lentil is limited. We used next-generation sequencing (NGS) technology to develop SSR markers in lentil. NGS allowed us to identify regions of the lentil genome that contained SSRs. Illumina Hiseq-2000 sequencing of the lentil genotype “Karacadağ” resulted in 1,727,734 sequence reads comprising more than 48,390 Mb, and contigs were mined for SSRs, resulting in the identification of a total of 8697 SSR motifs. Among these, dinucleotide repeats were the most abundant (53.38%), followed by trinucleotides (30.38%), hexanucleotides (6.96%), tetranucleotides (6.59%), and pentanucleotides (3.19%). The most frequent repeat in dinucleotides was the TC (21.80%), followed by the GA (17.60%). A total of 2000 primer pairs were designed from these motifs, and 458 SSR markers were validated following their amplified PCR products. A linkage map was constructed using these new SSRs with high linkage disequilibrium (209) and previously known SSRs (11). The highest number of SSR markers (43) was obtained in LG2, while the lowest number of SSR markers (19) was obtained in LG7. The longest linkage group (LG) was LG2 (86.84 cM), whereas the shortest linkage group was LG7 (53.46 cM). The average length between markers ranged from 1.86 cM in LG1 to 2.81 cM in LG7, and the map density was 2.16 cM. The developed SSRs and created linkage map may provide useful information and offer a new library for genetic diversity analyses, linkage mapping studies, and lentil breeding programs.

## 1. Introduction

Lentil (*Lens culinaris* L.) is one of the most important legume crops after beans and chickpea. It requires relatively low maintenance and has a wide range of adaptabilities from arid to temperate regions. As a rotation crop with cereals, lentil can fix nitrogen from the air, which boosts soil fertility and decreases the demand for synthetic nitrogen fertilizers [1,2,3,4]. Apart from being an essential protein source and rotation crop, lentil offers numerous health benefits. Lentil is rich in dietary fiber, which promotes digestive health and the lowering of cholesterol levels; it is also rich in essential nutrients such as iron, potassium, folate, and magnesium, which help prevent anemia. Moreover, lentil has low fat and calorie contents, making them ideal for maintaining body weight within the ideal range [5]. Even though lentil is one of the most climate-resilient legume species, it has not received much attention compared to other legumes such as common beans and chickpea. Compared to chickpea, the development and application of cutting-edge tools such as molecular markers in lentil breeding are still limited. In relation to this, Coyne et al. [6] suggested that although the GenBanks have a significant number of lentil accessions, only a limited number of lentil genotypes have been used in the breeding approaches for years. Therefore, there is a need for germplasm screening using practical approaches such as genetic markers.

Advances in DNA technology have led to the development of DNA molecular markers for all crops [7]. Over the last few decades, studies of crop genomes have accelerated the development of new molecular markers. Molecular markers are quick and cost-effective tools for allele mining and marker-assisted selection (MAS), especially in crops with large genomes [4,8,9,10,11,12]. Lentil has a relatively large genome (4 GB), and various molecular markers have been used for the evaluation of genetic diversity in lentil [13,14,15,16,17,18,19,20]. Simple sequence repeat (SSR) markers show high polymorphism and reproducibility and are very useful and practical compared to other marker types [21,22].

SSR markers are essential tools for lentil breeding because of their potential for genetic diversity analysis, MAS, and genetic mapping, which can aid in the development of new varieties with desirable traits. One of the first genomic libraries for SSR markers in lentil was constructed using the *Sau3AI* enzyme, and 30 polymorphic SSR markers covering the lentil genome were developed using this library [23]. Later, 14 SSR markers [9] were used for the genetic characterization of the lentil collections. Verma et al. [24] created a library of 122 SSR primer pairs from the genomic library enriched with the GA/CT motif for use in lentil breeding programs. Andeden et al. [25] designed a library of 360 SSR primer pairs using four genomic libraries constructed from a local lentil (Karacadağ) genotype. A total of 47 out of 360 polymorphic SSRs were used to construct a genetic linkage map of the Karacadağ × Silvan F_2_ hybrid population. Recently, 53 SSR primer pairs from a genomic library enriched with the AG/AC motif were developed for use in lentil molecular characterization [26].

Genetic mapping is an essential prerequisite for MAS breeding, and although its use in major crops is widespread, the limited scope of marker development studies has resulted in a lack of genetic maps for lentil. One of the first lentil genetic maps was constructed by genotyping the F_2_ mapping population in the *L. culinaris* × *L. orientalis* hybrid using RFLP markers [27]. Most of the genetic maps made for lentil have been intra- and interspecies, and these genetic maps were constructed mostly using AFLP, RAPD, and RFLP markers [2,23,28,29,30,31,32,33,34,35,36,37]. The advent of next-generation sequencing (NGS) technology has facilitated lentil genotyping using SNP markers, opening new avenues for genomic research [38,39]. In addition to the construction of high-density genome maps, gene/QTL analysis, and marker-assisted selection, SSRs are suitable for functional and comparative genomics [40,41]. However, the development of SSRs is time-consuming and expensive. Traditionally, a large number of primer pairs have been screened to develop a limited number of polymorphic SSR markers. The identification of SSR markers now requires much less work and is more affordable due to developments in NGS technologies [42]. Although some SSR markers have been developed in lentil over the years [21,23,43], when compared to the other pulse crops, coverage of currently available SSR markers is very limited for the analysis of genetic diversity in a species with a relatively large genome and worldwide distribution [21,23,43]. To address this gap, we utilized next-generation sequencing technology (1) to develop new SSR markers in lentil and (2) to construct a genetic linkage map using these markers.

## 2. Materials and Methods

### 2.1. Plant Material

Karacadağ genotypes from Southeast Turkey (selected from local landraces) were used to develop SSR markers. A recombinant inbred line (RIL) population of 91 genotypes was generated using Karacadağ and Silvan genotypes. Andeden et al. [25] provided comprehensive information on the generation of the RIL mapping population. Single-seed descent was used to create recombinant inbred lines, which involved selfing the F2 generation for an extra six generations to produce the F8 generation.

### 2.2. DNA Isolation and Sequencing

Genomic DNA was extracted from the parents (Karacadağ and Silvan) and 91 RILs using the standard CTAB protocol described by Doyle and Doyle [44]. The quality and quantity of the DNA samples were assessed using a spectrophotometer (GeneQuant Pro, Amersham Biosciences; Little Chalfont, Buckinghamshire, UK). DNA was diluted to 10 ng/μL for use in SSR applications. Genomic DNA was extracted from fresh leaves of the Karacadağ genotypes to send to BGI (Beijing Genomic Institute, Hong Kong, China) for sequencing on the Illumina HiSeq-2000 platform to develop SSR markers. The Illumina Hiseq-2000 sequencing system worked as follows: the genomic DNA was fragmented and run in the gel. After electrophoresis, DNA fragments of the desired length were purified from the gel. Then, adapter ligation and DNA cluster preparation were performed and subjected to Hiseq-2000 sequencing.

### 2.3. SSR Detection, Annotation, and Primer Design

The development of SSR primer pairs for lentil involved the utilization of DNA sequences with 5× coverage of the “Karacadağ” genotype obtained from Illumina HiSeq-2000 sequencing. Short sequences were assembled using SOAPdenovo (at http://soap.genomics.org.cn/soapdenovo.html, accessed on 24 September 2013), and SSRIT software (http://www.gramene.org/db/markers/ssrtool, accessed on 24 September 2013) was used to identify repeated motifs in the assembled sequences. SSR primer design utilized repetition sequences of 20 bases or more, with a minimum threshold of 10 repeats for dinucleotides, 7 repeats for trinucleotides, 5 repeats for tetranucleotides, and 4 repeats for penta- and hexanucleotides. In addition, 7 repeats for dinucleotides, 5 trinucleotides, 4 tetranucleotides, and 3 penta-and hexanucleotides were also included in the study. SSR primer design was conducted using the web-based BatchPrimer3 v1.0 software [45] with standard parameters and some modifications (max mispriming: 8; pair max mispriming: 16; min% GC: 40; max self-complementarity: 6; max 3′ self-complementarity: 2; and max poly X: 4).

### 2.4. Validation and Selection of SSR Primer Pairs

The annealing temperatures of the SSR primer pairs were determined based on primer sequence information. To determine polymorphism levels and validate the identified SSR primer pairs, pre-screening was first carried out in the Karacadağ and Silvan genotypes.

In the SSR analysis, capillary electrophoresis was employed for all electrophoresis procedures using an ABI 3130xl automatic genetic analyzer (Applied Biosystems, Foster City, CA, USA). The PCR reactions and cycle conditions were performed using a universal M13 primer following Schuelke [46]. Advanced primers were synthesized by adding the 5′-TGTAAAACGACGGCCAGT-3′ M13 universal primer to the 5′ ends. At the same time, the M13 primer was synthesized by labeling it with a 5′ end and 6-FAM, VIC, NED, and PET fluorescent dyes. The SSR reactions included a reverse primer, a forward primer with an M13 primer added to the 5′ end, and a labeled M13 primer.

SSR amplification was carried out using a 12.5 µL PCR reaction mixture containing 75 mM of Tris-HCl; 20 mM of (NH4)_2_ SO4; 2.0 mM of MgCl_2_; 0.01% Tween 20; 200 mM of dNTP; a forward primer with M13 universal primer added to the 5’ end (average 40 bases) at a concentration of 20 nM; a reverse primer at a concentration of 200 nM (average 20 bases); an M13 universal primer labeled with FAM, VIC, NED, or PET at a concentration of 200 nM; 0.7 units of Taq DNA polymerase; and 10–20 ng of DNA at a final pH of 8.8. The PCR included an initial denaturation step for 5 min at 94 °C, followed by 28 cycles of denaturation at 94 °C for 45 s, primer annealing at 50–60 °C (depending on the primer pair) for 45 s, and extension at 72 °C for 1.5 min. For the first eight cycles, denaturation was carried out for 45 s at 94 °C, annealing for 45 s at 52 °C, extension for 1.5 min at 72 °C, and a final extension cycle at 72 °C for 5 min. After the PCR procedure, 0.5 µL of PCR product was mixed with 9.8 µL of Hi-Di Formamide (Applied Biosystems, Waltham, MA, USA) and 0.2 µL of LIZ-500 size standard and denatured for 5 min at 95 °C, chilled on ice, and separated using the ABI 3130xl Genetic Analyzer software v3.7 (Applied Biosystems, Foster City, CA, USA) to determine fragment sizes via Genemapper following the user manual.

### 2.5. Construction of Genetic Linkage Maps

A total of 458 newly generated and 59 previously reported SSRs [25] were tested on two lentil genotypes (Karacadağ and Silvan). The markers that passed the criteria (209 + 11) were involved in the construction of the linkage map. JoinMap v5 software [47] was used to construct genetic linkage groups. The lengths and recombination units of the linkage groups were calculated in centimorgan (cM) according to Kosambi [48]. To test the conformity of the markers to the expected “1:1” segregation ratio, chi-square (*X*^2^) values for each marker with the “Locus genotype frequency” function command in JoinMap software were calculated, and markers that deviated from the expected Mendel ratios were determined. To ensure the optimum distribution of the markers on the linkage groups by placing the least deviating markers in the connection groups, the average values of the linkage groups were calculated with the “Average chi-square contribution” function command. The construction of the linkage map was done with “LOD grouping” using the Kosambi mapping function [48]. To determine the marker order in each linkage group, standard settings of the JoinMap software were used, and for the best marker order, the calculations were repeated three times. Finally, the arrangement of the linkage groups and graphical placements was performed using MapChart 2.2 software [49].

## 3. Results

### 3.1. Sequence Assembly and Simple Sequence Repeat Identification

The local landrace Karacadağ was sequenced on the Illumina HiSeq-2000 platform for the development of a library and identification of SSR markers. Initial sequencing resulted in 1,727,734 reads comprising more than 48,390 Mb. After removing the adaptors from sequence reads, the final size was 47,200 Mb with an average cleaned sequence length of 164.8 nucleotides (nts).

**Figure 1:**
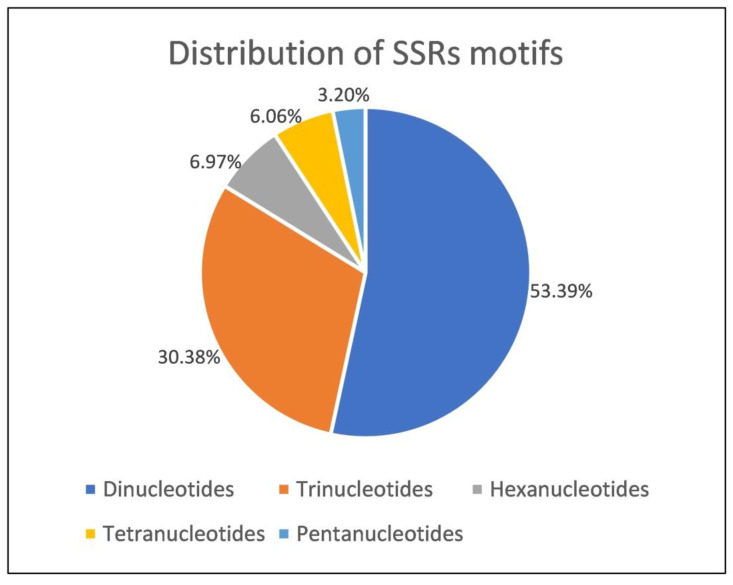
Distribution of simple sequence repeat (SSR) motifs in lentil genome.

To identify new SSRs and create an SSR library for the lentil genomic studies, we screened initial sequence reads and identified a total of the 8697 SSRs with lengths ranging between 6 and 32 nts with an average length of 13.9 nts. The identified SSRs were various in length as di, tri, tetra, penta, and hexanucleotides. The most abundant repeat motif was dinucleotides with a 53.38% ratio among all SSRs. Trinucleotide repeats were the second most common type of repeats (30.38%), followed by hexanucleotide repeats (6.96%). The least common repeat types were tetranucleotides (6.59%) and pentanucleotides (3.19%) (Table 1 and Figure 1).

**Figure 2:**
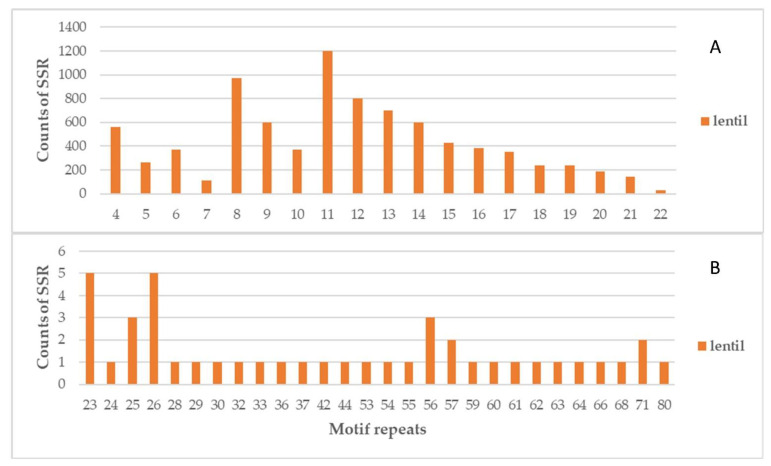
Distribution of the number of SSRs for per-repetitions of the motifs in lentil genome. The plot is divided into two separate plots. Plot (**A**) demonstrates the highest number of SSR motifs, whereas Plot (**B**) shows the lowest number of SSR motifs.

The entire sequence data were searched for the SSR repeat motifs starting with the four or more repeats. According to the results, the top three most frequent motifs for SSRs were 11, 8, and 12 repeats. These particular motifs accounted for a significant portion of the initial 8697 identified SSRs. (Figure 2). As mentioned above, only SSRs with a minimum of 20 bp and 10 or more repeats were used for the primer design. The most common repeat motif was TC (21.80%), followed by GA (17.60%), AG (15.7%), CT (12.60%), and AT (10.90%). In contrast, TTA (10.80%) was the most common motif in trinucleotides, followed by AAT (10.60%). The most common motif for tetranucleotides was AAAT (15.10%) (Table 2).

### 3.2. Primer Design and SSR Validation

**Figure 3:**
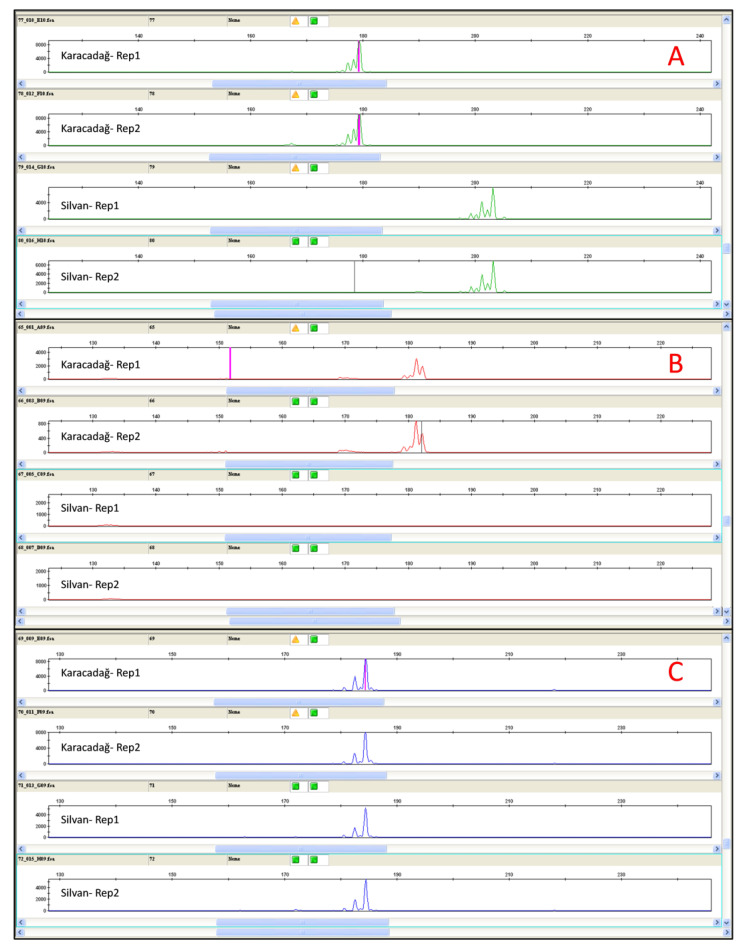
Peak examples that appeared during SSR validation processes for the parental genotypes Karacadağ and Silvan: (**A**) showed peaks on both genotypes; (**B**) showed a peak on only one genotype (Karacadağ or Silvan) and did not fit SSR character; (**C**) monomorphic markers.

The data obtained from the Illumina HiSeq-2000 platform for the single genotype Karacadağ were used for the search and identification of SSR markers. Of the 8697 SSR contigs, 2000 were selected and used for marker design. The amplification efficiencies of the designed 2000 markers were tested on two lentil genotypes, Karacadağ and Silvan, which were also parents of the mapping population. As a result of the PCR amplification products, 458 (22.90%) markers showed scoreable peaks in capillary electrophoresis. Out of 458 primer pairs, 310 displayed polymorphic peaks for both genotypes, as expected for the co-dominant character of SSR markers (Figure 3A), whereas 40 primer pairs demonstrated the peaks for only one genotype (Karacadağ or Silvan) and acted as dominant markers that did not fit the SSR markers’ character (Figure 3B). Furthermore, of the remaining markers, 108 were monomorphic and therefore unsuitable for further analysis (Figure 3C). The details of the designed SSR primers are given in Table S1.

### 3.3. Construction of Genetic Linkage Map

**Figure 4:**
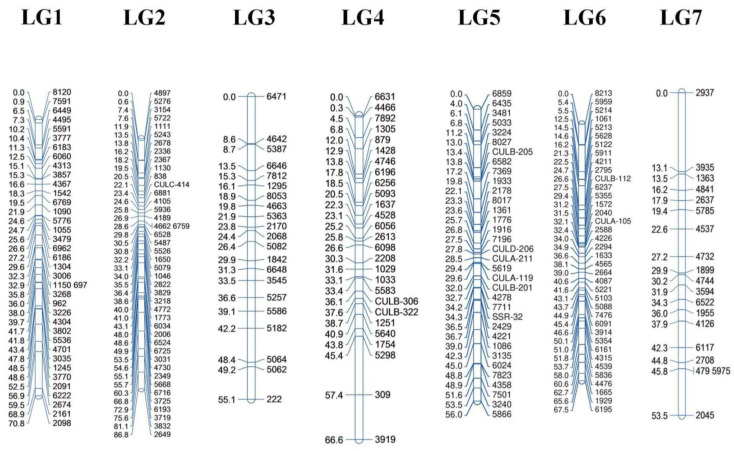
The genetic linkage map of the *Lens culinaris* RIL population obtained via the cross of Karacadağ × Silvan genotypes. The marker positions at cM length are demonstrated on the left side of the LG bars, whereas the marker names are shown on the right side of the bars. The names of the newly developed SSRs include only numbers, while the previously known marker names include word–number patterns.

A total of 458 newly created SSR markers passed the criteria expected from an SSR marker; however, in the end, 310 scoreable and polymorphic markers remained to be used in the construction of the genetic map. In addition to the newly generated SSRs, 59 SSRs previously reported by Andeden et al. [25] were used to increase the resolution of the genetic map and confirm the linkage groups. These markers were also involved in the elimination process together with new SSRs. After the initial steps of eliminating SSRs with high linkage disequilibrium, the final analysis yielded a genetic map consisting of 220 SSRs (209 new and 11 previously identified), which were found to be distributed across seven linkage groups (LG1, LG2, LG3, LG5, LG6, and LG7) of the lentil genome (Table 3, Figure 4). 

The resulting linkage map exhibited variations in the linkage groups (LGs): different numbers of markers were distributed across the LGs at varying densities. In this context, the highest number of SSR markers was in LG2 with 43 markers, and the lowest was in LG7 with 19 markers. Accordingly, the highest percentage distribution of the markers was in LG2 (19.55%), and the lowest was in LG7 (8.64%). The map length per chromosome followed the same trend as the marker distribution: LG2 had the longest map length at 86.84 cM, and LG7 had the shortest map at 53.46 cM. The average marker distance ranged between 1.86 cM (LG1) and 2.81 cM (LG7), whereas the mean density for the whole map was 2.16 cM. The maximum gap between two adjacent markers was 13.11 cM on LG7, which was expected when considering that it had the lowest number of markers and density among the LGs. LG5 had the smallest maximum gap length among all LGs with an average marker distance of 4.41 cM (Table 3).

## 4. Discussion

Plant breeding has reached the era of fast, effective, and reliable data analyses. Due to the fast pace of climate change, population increase, and unstoppably evolving pests and diseases and abiotic stress factors, there is a continuous need for the identification and validation of novel genetic diversity [6,50,51,52]. Single-nucleotide polymorphism (SNP) and simple sequence repeat (SSR) markers are two of the most popular and practical approaches used to dissect genetic diversity [38,53,54,55]. The use of NGS for allele mining and sequencing has become convenient and cost-effective [42]. It has practical outcomes and provides a vast amount of sequence data at a relatively low cost. Here, we used the NGS approach for the identification of novel SSR markers in the lentil genome.

### 4.1. Identification of Novel SSR Markers

SSRs are considered important tools for genetic diversity, population structure, determination of phylogenetic relationships, generation of linkage maps, QTL mapping, and marker-assisted selection [38,56,57]. Here, the NGS approach provided a large data set for an SSR library. As a result of the sequencing, we identified 8697 repetitive chromosomal regions. A large portion of these regions contained dinucleotide repeats (53.40%). When the motif types were analyzed, TC repeats were found to be the most common repeat motif, followed by GA motifs. After the initial steps of cleaning and filtration, 2000 repetitive regions were randomly selected and evaluated as SSR candidates. After PCR amplification and polymorphism detection across 2000 selected markers among 8697, we found that 458 SSRs met the usability criteria. From this collection, we used a total of 209 newly generated polymorphic SSRs and 11 SSRs previously known that were selected according to their linkage disequilibrium levels for linkage mapping. Despite the limited number of studies on the identification of new SSR markers using NGS tools, several studies have been published on lentil. Bakir and Kahraman [26] used enriched genomic libraries rich in AC and AG repeats to construct new SSR markers. They used the cv. Kafkas-obtained 350 clones, resulting in 53 newly developed SSRs. According to their results, the most common motifs were GA/CT (62.60%), and most of the repeats were dinucleotide repeats. In a similar study, Singh et al. [56] identified 9949 EST-SSR loci from the RNA-Seq data and validated 50 SSRs. Mononucleotide repeats were the most abundant (51.00%), followed by the trinucleotide repeats with a 30.00% ratio. Verma et al. [58] mined lentil transcripts to find genic SSR markers. They obtained 8722 SSR candidate regions; trinucleotide repeats were the most common (54.72%), followed by tetranucleotide repeats (20.06%). The most common motif was AAG/CTT repeats (13.12%). They randomly selected 96 SSRs from the library, of which 82 were polymorphic in the parent genome. These markers may provide useful information within the Lens genera as well as other legume species with cross-genera marker transferability [11]. Genomic-DNA-based SSR markers would enhance our ability to screen germplasm accessions, mapping populations, and cultivars for various purposes [38]. A large number of markers are required to increase the depth and resolution of genetic maps and enable studies at the gene level, and it is essential to have more SSRs globally available and practically usable.

### 4.2. Construction of Genetic Linkage Map Using New SSR Markers

Lentil has a large genome of about 4 GB, and genetic and genomic studies are not easily conducted due to the genome size and complexity. Genetic mapping is still a reliable and practical approach for understanding the mechanisms of traits of interest and for marker-assisted selection (MAS) using chromosome-tagged SSRs. Here, 209 newly developed and 11 previously known SSR markers were used for the construction of the lentil linkage map. The total map length was 456.33 cM, while the mean marker distance was 2.16 cM. To evaluate our map, we compared it with maps previously reported in lentil. In this context, in a mapping study with RAPD, ISSR, and SSR markers, Gupta et al. [2] used an F_2_ population and created a map with 199 markers covering a map length of 3843.40 cM and 19.30 cM marker distance in lentil. In another mapping study, Kaur et al. [33] used a RIL population and constructed a linkage map with 57 SSR and 261 SNP markers distributed onto 10 LGs covering a total map length of 1178 cM and a 3.70 cM marker density. Verma et al. [59] used a RIL population and built a linkage map with a total of 216 SSR markers with a total map length of 1183.70 cM and a 5.48 cM average marker density. Compared to our observations, these maps were quite long in terms of map length but had a higher marker–marker interval. These differences may be due to the different marker types and population structures. A Karacadağ × Silvan population was previously used for linkage mapping by Andeden et al. [25]; however, it was at the F2 stage. In this old version of the map, 43 SSRs spanned 303.90 cM with a 7.06 cM average marker density of the linkage groups. LG2 was the longest among all the LGs, whereas LG7 was the shortest. The largest gap was observed for LG3 with a length of 25.50 cM. The current version of the map used the same parental population but at the F_8_ stage and added 209 entirely new markers to the map. In this study, we produced a longer and denser map than in the previous version. It is important to note that in this version, the shortest and longest linkage groups remained the same as those in the previous versions. The maximum gap interval on the linkage groups decreased considerably from 25.50 to 13.11 cM in LG7. Based on previous maps and the old and new versions of the Karacadağ × Silvan map, it can be concluded that the RIL population used in this study is well suited for linkage mapping, and the mapped markers provide better coverage than previous versions. When we compared our results for the 11 previously known SSRs with Andeden et al. [25], we found quite similar positions in 9 out of 11 SSRs. According to the comparison, SSR markers CULD206, CULA119, CULB201, and SSR32 were positioned in the same linkage group. Similarly, CULB112 and CUL105 were also in the same linkage group in this study and that by Andeden et al. [25]. CULC414 was on a separate linkage group in this study and that by Andeden et al. [25]. The preservation of the positions of the SSRs obtained in previous studies can be considered a validation of the reliability of the SSRs used in a given study.

Even though the initial cost of SSR development is not cheap and is time-consuming, end-user benefits include ease of use, global applications, and usage in almost all areas of plant breeding and other related disciplines. Therefore, once created and made publicly available, these SSR libraries would have a global audience. Since the production and consumer markets of lentil are mostly developing countries, SSR-based MAS breeding solutions would enhance their breeding capacity and speed up processes.

## 5. Conclusions

Lentil is known as one of the most important protein sources in developing countries among legumes. It is important to note that lentil is a significant source of protein and can contribute to addressing nutritional problems caused by protein deficiency. Rapid, effective, and result-oriented breeding studies should be carried out to improve the yield and quality characteristics of lentil. Indeed, the use of marker-assisted selection studies in lentil has the potential to significantly contribute to the development of lentil varieties with higher protein content and other desirable traits, thereby addressing nutritional problems caused by protein deficiency. Therefore, it is necessary to identify, validate, and present new, unique, and a high number of markers for the use of breeders. The outputs of this study provided 8697 SSR motifs, 220 polymorphic SSR markers, and a 456.33 cM long lentil genetic linkage map, which may serve as a reference library for future studies. The combination of these findings could potentially provide valuable insights for genetic and genomic studies in lentil, particularly in relation to lentil breeding strategies.

## Figures and Tables

**Table 1:** Simple sequence repeat types in lentil genome.

Type of SSRs	Number of SSRs	%
Dinucleotides	4643	53.38
Trinucleotides	2643	30.38
Tetranucleotides	527	6.59
Pentanucleotides	278	3.19
Hexanucleotides	606	6.96
Total	8697	~100.00

**Table 2:** Abundantly identified SSR motifs in the genome of the Karacadağ genotype.

^a^ SSR Motifs	Number of SSRs (F ≥ 10)	Motif Frequency (%)
AG	727	15.65
AT	507	10.91
CT	587	12.64
GA	815	17.55
TC	1013	21.81
AAT	279	10.55
TTA	285	10.78
AAAT	80	15.18

^a^ Only frequencies higher than 10% of fragments were demonstrated.

**Table 3:** Distribution of the 220 genomic SSR markers on the seven linkage groups of an intraspecific linkage map of lentil.

Linkage Groups	No. of SSRs	Pct. of SSRs (%)	Length (cM)	Marker Density (cM) ^a^	Max. Gap Length (cM) ^b^
LG1	38	17.27	70.79	1.86	9.36
LG2	43	19.55	86.84	2.01	6.73
LG3	21	9.55	55.13	2.62	8.64
LG4	27	12.27	66.61	2.46	11.92
LG5	34	15.45	56.00	1.64	4.41
LG6	38	17.27	67.50	1.77	7.07
LG7	19	8.64	53.46	2.81	13.11
Total-Mean	220	100.00	456.33	2.16 *	8.74 *

^a^ The ratio of length (cM) and number of SSRs per LG; ^b^ the maximum distance between two adjacent markers, * the mean of marker density and maximum gap length.

## Data Availability

The sequence of SSR primer pairs can be obtained from Table S1.

## Supplementary Materials

The following supporting information can be downloaded at: https://www.mdpi.com/article/10.3390/life13071579/s1, Table S1: Primer list
